# Biomarkers in the Rat Hippocampus and Peripheral Blood for an Early Stage of Mental Disorders Induced by Water Immersion Stress

**DOI:** 10.3390/ijms24043153

**Published:** 2023-02-05

**Authors:** Keisuke Suzuki, Junko Shibato, Randeep Rakwal, Masahiko Takaura, Ryotaro Hotta, Yoshinori Masuo

**Affiliations:** 1 Laboratory of Neuroscience, Department of Biology, Faculty of Science, Toho University, 2-2-1 Miyama, Funabashi 274-8510, Japan; 2 Department of Functional Morphology, Shonan University of Medical Sciences, 16-48 Kamishinano, Totsuka-ku, Yokohama 244-0806, Japan; 3 Institute of Health and Sport Sciences and Tsukuba International Academy for Sport Studies (TIAS2.0), University of Tsukuba, 1-1-1 Tennodai, Tsukuba 305-8574, Japan

**Keywords:** mental disorder, corticosterone, anxiety, rat, hippocampus, peripheral blood, MKP-1, CEBPD, MMP-8

## Abstract

It is difficult to evaluate the pre-symptomatic state of mental disorders and prevent its onset. Since stress could be a trigger of mental disorders, it may be helpful to identify stress-responsive biomarkers (stress markers) for the evaluation of stress levels. We have so far performed omics analyses of the rat brain and peripheral blood after various kinds of stress and have found numerous factors that respond to stress. In this study, we investigated the effects of relatively moderate stress on these factors in the rat to identify stress marker candidates. Adult male Wistar rats underwent water immersion stress for 12 h, 24 h, or 48 h. Stress caused weight loss and elevated serum corticosterone levels, and alterations regarded as anxiety and/or fear-like behaviors. Reverse-transcription PCR and Western blot analyses revealed significant alterations in the expressions of hippocampal genes and proteins by the stress for no longer than 24 h, such as *mitogen-activated protein kinase phosphatase 1 (MKP-1)*, *CCAAT/enhancer-binding protein delta (CEBPD)*, *small ubiquitin-like modifier proteins 1/sentrin-specific peptidase 5 (SENP5)*, *matrix metalloproteinase-8 (MMP-8)*, *kinase suppressor of Ras 1 (KSR1),* and MKP-1, MMP-8, nerve growth factor receptor (NGFR). Similar alterations were observed in three genes (*MKP-1*, *CEBPD*, *MMP-8*) in the peripheral blood. The present results strongly suggest that these factors may serve as stress markers. The correlation of these factors in the blood and brain may enable the evaluation of stress-induced changes in the brain by blood analysis, which will contribute to preventing the onset of mental disorders.

## 1. Introduction

Stress may cause mental disorders, such as depression [1,2]. Most of the previous studies focused on treatment for patients with psychiatric disorders [3,4], and assessing pre-symptomatic status remains difficult. During the pathogenesis of mental disorders, stress increases the release of glucocorticoids by activation of the hypothalamic–pituitary–adrenocortical axis, leading to atrophy and inhibition of neurogenesis in the hippocampus [5,6]. Indeed, patients with depression exhibit a marked decrease in hippocampal volume [7] and function [8]. Chronic mild stress (CMS) has been demonstrated to cause functional impairments, particularly learning disabilities and memory impairment in rats [9]. To evaluate stress levels, several kinds of physiological parameters have been suggested [10,11]. Attention has been focused on whole-body stress biomarkers in saliva and urine, such as heat stress markers, innate immune markers such as acute phase protein (APP), oxidative stress markers, and chemical secretions [12,13].

Depressive behaviors can be seen in experimental animals through several types of mild stress, such as water immersion stress [14,15]. Stress causes a decrease in brain-derived neurotrophic factor (BDNF) expression in the hippocampus [16], and antidepressant drugs increase the expression in the limbic system [17]. Similarly, superoxide dismutase and catalase activities in the rat hippocampus were reduced by stress, while treatment with lamotrigine, an anticonvulsant and putative mood stabilizer, increased their activity in the amygdala [18]. Moreover, depression has a high degree of comorbidity with inflammatory diseases such as allergies, stroke, multiple sclerosis, rheumatoid arthritis, and encephalitis [19]. In fact, stress causes inflammation in the brain, leading to the development of diseases such as depression [20]. Stress increases the expression of pro-inflammatory cytokines [21] and decreases the expression of anti-inflammatory cytokines [22] in the rat brain. However, there are still many mental disorders for which the mechanisms that lead to their onset have not been elucidated. Recently, changes in glial cells have also attracted attention [23].

On the other hand, natural substances that have anti-stress effects have been known for a long time and have been used empirically, and their effects have recently been proven by alterations in the expression of intracerebral factors [24,25]. For example, the stress response is suppressed by coffee bean aroma, which may have antioxidant and anxiolytic properties that down-regulate the expression of peroxiredoxin (PRX) in the rat brain [26]. We also demonstrated the effects of several kinds of aroma, including sesame oil, suppressed stress responses in rodents at the level of behavior and gene/protein expression in the brain [24,27]. However, it is extremely difficult to objectively grasp the human mental state, and as a result, people tend to go through a pre-symptomatic state and develop a mental disorder without being aware of increased stress levels.

To realize the objective evaluation of the stress level, stress-responsive biomarkers (stress markers) should be identified. If alterations in its expression in the brain and peripheral blood correlate with stress, it may be possible to extrapolate changes in the brain by analyzing peripheral blood. In addition, various stress markers are also important to clarify the pathogenesis of stress-induced mental disorders. We recently performed transcriptome and proteome analyses of the rat brain and peripheral blood after several kinds of stress [24]. In total, significant changes were found in the expression of 135 and 871 genes in the brain and blood, respectively, by more than two kinds of stress. In the present study, we investigated the effects of water immersion stress on the expression of 6 proteins in the rat hippocampus. Moreover, gene expression was also measured not only in the hippocampus but also in the peripheral blood. We have selected eight genes, according to the annotation, out of 43 genes whose expression was altered in both brain and blood.

## 2. Results

### 2.1. Effects of Water Immersion Stress on Body Weight and Serum Corticosterone Levels

**Figure 1:**
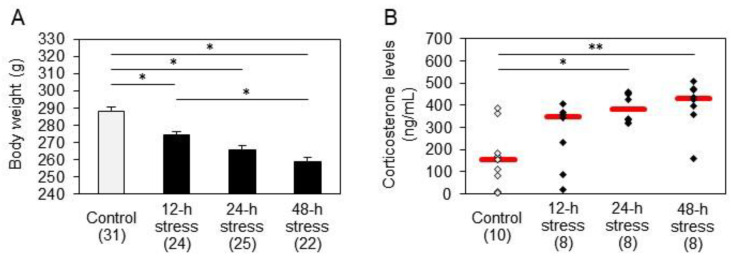
Effects of water immersion stress on rat body weight and serum corticosterone concentrations. (**A**) The rat body weight. Bars in the graph represent the mean ± S.E.M. * *p* < 0.01 by Tukey–Kramer test. (**B**) Corticosterone levels in the rat serum. Red bars represent the median. Parenthesis: the number of animals. * *p* < 0.05, ** *p* < 0.01 by Steel−Dwass test. Stress reduced body weight significantly, which was proportional to the duration of water immersion stress. Serum corticosterone levels were significantly high after 24 h and 48 h stress.

All stressed animal groups exhibited reduced body weight relative to that of the control animals, which was statistically significant (Figure 1A). This effect was proportional to the duration of water immersion stress. In addition, the body weight of the 48-hour stress group was significantly lower than that of the 12-hour stress group. Serum corticosterone levels were significantly higher in rats exposed to 24 h and 48 h water immersion stress than in control rats (Figure 1B). Serum corticosterone levels tended to increase even after 12 h of stress. However, the changes in corticosterone levels for this relatively short stress showed large variability and were not statistically different. However, changes in corticosterone levels due to this relatively short duration of stress were highly variable and not statistically different from controls.

### 2.2. Behavioral Changes after Water Immersion Stress

**Figure 2:**
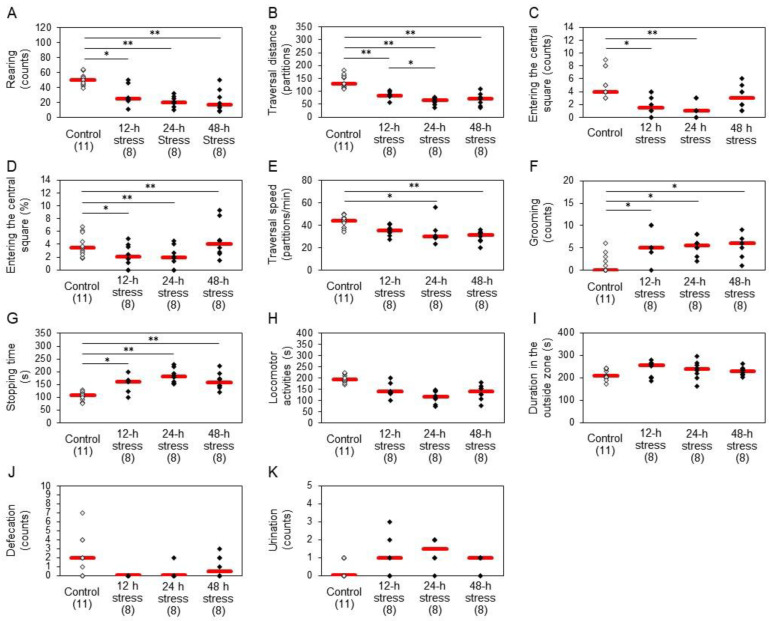
Effects of water immersion stress on rat behavior in the open field test. (**A**) Rearing; (**B**) traversal distance, (**C**) entering the central square (counts); (**D**) entering the central square (%); (**E**) traversal speed; (**F**) grooming; (**G**) stopping time; (**H**) locomotor activities; (**I**) duration in the outside zone; (**J**) defecation; (**K**) urination. Red bars represent the median. Parenthesis: the number of animals. * *p* < 0.05, ** *p* < 0.01 by Steel−Dwass test. Stress caused anxiety-like symptoms, such as significant decreases in rearing, travel distance, entering the central square, and travel speed, although the percentage of entering the central square was significantly increased in 48 h stress rats. Stress also induced a significant increase in grooming and stopping time. Alterations in entering the central square and stopping time in 24 h stress group seemed maximal.

In the open field test (Figure 2), all stressed groups exhibited a variety of anxiety-like symptoms, including significant decreases in rearing behavior (Figure 2A), travel distance (Figure 2B), entering the central square (Figure 2C,D), and travel speed (Figure 2E). Among these, a significant increase was found in the percentage of entering the central square only after 48 h stress (Figure 2D). Stress-treated animals showed significant increases in grooming (Figure 2F) and stopping time (Figure 2G). Alterations in entering the central square (Figure 2C,D) and stopping time (Figure 2G) in the 24 h stress group seemed maximal, and changes in the 48 h stress group were opposite to those in the 12 h and 24 h stress groups. Significant changes were not observed in locomotor activities (Figure 2H) and duration in the outside zone (Figure 2I). The defecation counts seemed lower in the stressed rats than in control animals (Figure 2J), and these rats urinated more frequently than controls (Figure 2K), although the differences were not statistically significant.

**Figure 3:**
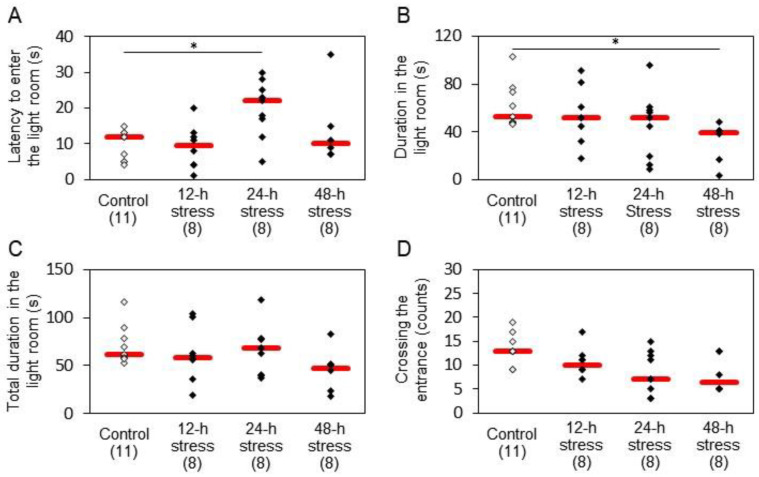
Effects of water immersion stress on rat anxiety-related behavior in the light/dark box test. (**A**) Latency to enter the light room; (**B**) duration in the light room; (**C**) total duration in the light room; (**D**) crossing the entrance. Red bars represent the median. Parenthesis: the number of animals. * *p* < 0.05 by Steel−Dwass test. Stress for 24 h caused an increase in the latency to enter the light room, and 48 h stress reduced the time spent in the light room, both of which were statistically significant.

In the light/dark box test, 24 h stress caused an increase in the latency to enter the light room, which was statistically significant (Figure 3A). The time spent in the light room was significantly reduced by 48 h stress (Figure 3B). Significant changes were not observed in the total duration in the light room (Figure 3C) and the crossing of the entrance (Figure 3D).

### 2.3. Protein Expression Profiles in the Hippocampus

**Figure 4:**
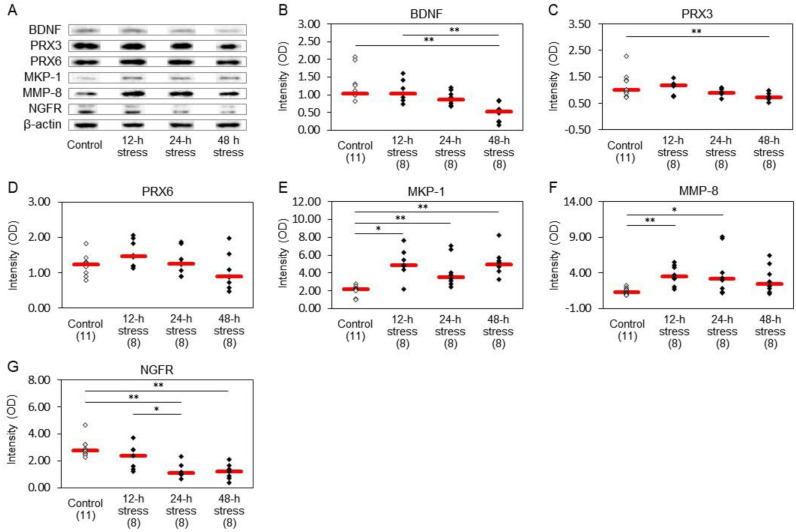
Protein expression profiles in the rat hippocampus. After exposure to stress, the hippocampi were immediately dissected, placed on ice, and protein expression was determined by Western blotting. The intensities of optical densities are expressed relative to beta-actin expression in each sample. (**A**) Representative blots of BDNF, PRX3, PRX6, MKP-1, MMP-8, NGFR, and beta-actin. (**B**) BDNF; (**C**) PRX3; (**D**) PRX6; (**E**) MKP-1; (**F**) MMP-8; (**G**) NGFR. Red bars represent the median. Parenthesis: the number of animals. * *p* < 0.05, ** *p* < 0.01 by Steel−Dwass test. BDNF expression was significantly decreased after 48 h stress compared to the control and 12 h stress groups. Stress for 12 h and 24 h caused significant increases in MKP-1 and MMP-8. MKP-1 expression was also significantly high after 48 h stress. On the other hand, NGFR expression showed significant decreases after 24 h and 48 h stress.

Western blots revealed alterations in the expression of several kinds of protein (Figure 4A). The expression of beta-actin in the rat hippocampus was stable and did not affect the alteration levels of other proteins under our experimental conditions. Notably, decreased expression of BDNF was observed in the 48 h stress group compared to those of the control and 12 h stress groups (Figure 4B). Further, 48 h water immersion stress caused a significant decrease in PRX3 (Figure 4C) but not in PRX6 (Figure 4D) protein expression. These factors have been suggested to respond to stress as described in the introduction.

We also measured the expression of mitogen-activated protein kinase (MAPK) phosphatase 1 (MKP-1), matrix metalloproteinase-8 (MMP-8), and NGFR because of their high priority as stress marker candidates as a result of gene expression analysis performed in the present study. We found increased levels of MKP-1 (Figure 4E) and MMP-8 (Figure 4F) protein expression after 12 h and 24 h stress. The expression of MKP-1 was also significantly increased after 48 h stress (Figure 4E). On the other hand, a significant decrease was found in nerve growth factor receptor (NGFR) expression after 24 h and 48 h stress (Figure 4G). Rats stressed for 48 h exhibited higher expression of MKP-1 and lower expression of BDNF, PRX-3, and NGFR than control rats. After 24 h stress, the expression of MKP-1 and MMP-8 were significantly higher, but NGFR was lower than the control.

### 2.4. Gene Expression Profiles in the Hippocampus and Peripheral Blood

**Figure 5:**
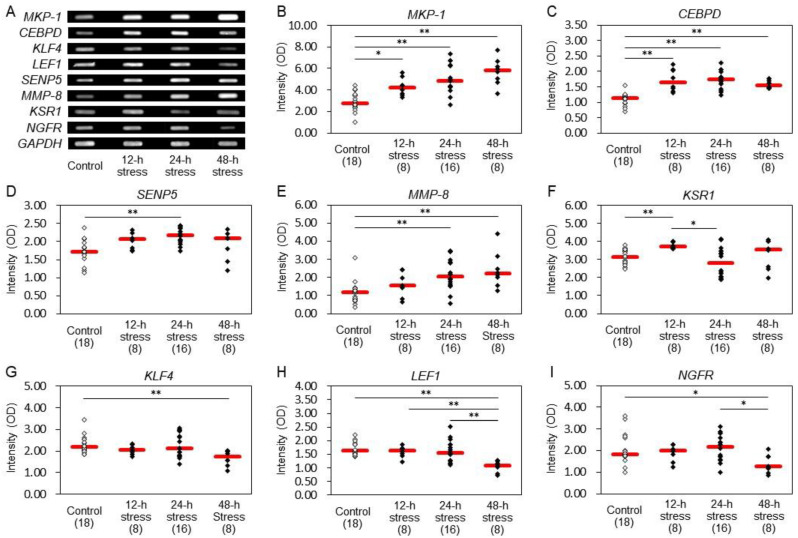
Gene expression profiles in the rat hippocampus. After exposure to stress, the hippocampus was immediately dissected, placed on ice, and mRNA expression was determined by RT-PCR. In preliminary experiments, we clarified the PCR cycle number at which the expression level of each gene saturated, and we adopted the PCR cycle number lower than the gene expression level saturating in the present study. The OD obtained from each sample was verified to be within the range in which the relationship between the amount of DNA and its OD was linear. (**A**) Representative gene expression bands of 8 kinds of genes and *glyceraldehyde 3-phosphate dehydrogenase* (*GAPDH*) by electrophoresis after RT-PCR. (**B**) *MKP-1*; (**C**) *CCAAT/enhancer-binding protein* (*C/EBP*) *delta*) (CEBPD); (**D**) *small ubiquitin-like modifier proteins* (*SUMO*)*1*/*sentrin-specific peptidase 5* (*SENP5*); (**E**) *MMP-8*; (**F**) *kinase suppressor of Ras 1* (*KSR1*); (**G**) *Krüpppel-like factor 4* (*KLF4*); (**H**) *lymphoid enhancer-binding factor 1* (*LEF1*); (**I**) *NGFR*. The intensities of optical densities are expressed relative to *GAPDH* expression in each sample. Red bars represent the median. Parenthesis: the number of animals. * *p* < 0.05, ** *p* < 0.01 by Steel−Dwass test. Stress for 12 h and/or 24 h stress caused a significant increase in the expression of *MKP-1*, *CEBPD*, *SENP5*, *MMP-8*, and *KSR1*. Stress for 48 h induced a significant increase in *MKP-1*, *CEBPD*, and *MMP-8*, with a decrease in *KLF4*, *LEF1*, and *NGFR*.

Electrophoresis after RT-PCR revealed alterations in the expression of several kinds of genes in the rat hippocampus (Figure 5A). The expression of *glyceraldehyde 3-phosphate dehydrogenase* (*GAPDH*) was stable and did not affect the alteration levels of other genes under our experimental conditions. The expression was significantly increased in *MKP-1* (Figure 5B), *CCAAT/enhancer-binding protein* (*C/EBP*) *delta* (*CEBPD*) (Figure 5C), *small ubiquitin-like modifier proteins* (*SUMO*)*1/sentrin-specific peptidase 5* (*SENP5*) (Figure 5D), *MMP-8* (Figure 5E), and *kinase suppressor of Ras 1* (*KSR1*) (Figure 5F) after 12 and/or 24 h stress. Similarly, increased expression of *MKP-1* (Figure 5B), *CEBPD* (Figure 5C), and *MMP-8* (Figure 5E) were found after 48 h, with decreased expression of *Krüpppel-like factor 4* (*KLF4*) (Figure 5G), *lymphoid enhancer-binding factor 1* (*LEF1*), (Figure 5H), and *NGFR* (Figure 5I).

**Figure 6:**
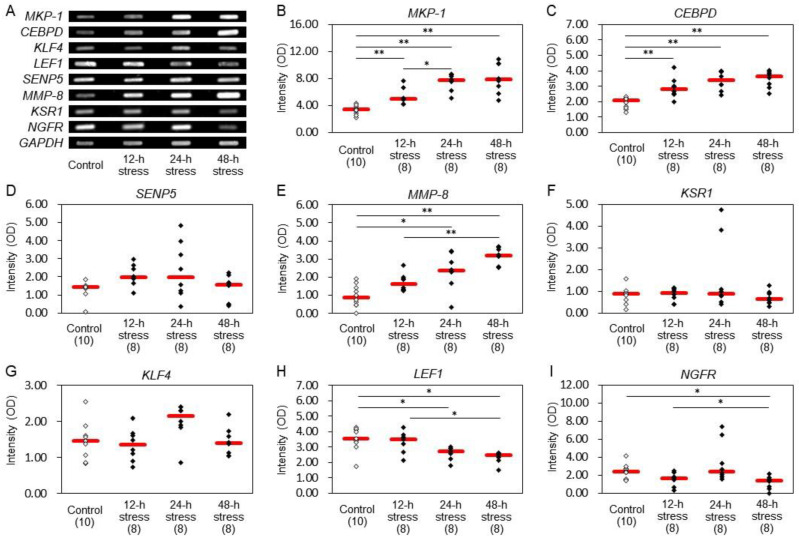
Gene expression profiles in the rat peripheral blood. After exposure to stress, blood was collected, and mRNA expression was determined by RT-PCR. The intensities of optical densities are expressed relative to *GAPDH* expression in each sample. In preliminary experiments, we clarified the PCR cycle number at which the expression level of each gene saturated, and we adopted the PCR cycle number lower than the gene expression level saturating in the present study. The OD obtained from each sample was verified to be within the range in which the relationship between the amount of DNA and its OD was linear. (**A**) Representative gene expression bands of 8 kinds of genes and *GAPDH* by electrophoresis after RT-PCR. (**B**) *MKP-1*; (**C**) *CEBPD*; (**D**) *SENP5*; (**E**) *MMP-8*; (**F**) *KSR1*; (**G**) *KLF4*; (**H**) *LEF1*; (**I**) *NGFR*. The intensities of optical densities are expressed relative to *GAPDH* expression in each sample. Red bars represent the median. Parenthesis: the number of animals. * *p* < 0.05, ** *p* < 0.01 by Steel−Dwass test. Stress for 12 h, 24 h, and 48 h induced a significant increase in the expression of *MKP-1* and *CEBPD*. *MMP-8* expression was significantly increased after 12 h and 24 h stress. On the other hand, the expression of *LEF1* was significantly decreased by 24 h and 48 h stress. After 48 h stress, *NGFR* expression was significantly decreased.

In peripheral blood, electrophoresis following RT-PCR was performed on eight genes as measured in the hippocampus. Comparatively, increased expression of *MKP-1* (Figure 6B), *CEBPD* (Figure 6C), and *MMP-8* (Figure 6E) and decreased expression of *LEF1* (Figure 6H) were observed in the peripheral blood samples of rats exposed to 12 and/or 24 h stress. In blood after 48 h stress, increased expression was found in *MKP-1* (Figure 6B), *CEBPD* (Figure 6C), and *MMP-8* (Figure 6E) and decreased expression in *LEF1* (Figure 6H) and *NGFR* (Figure 6I).

## 3. Discussion

In the present study, we found credible stress marker candidates by clarifying physiological, biochemical, and molecular changes due to stress. There is no doubt that biomarkers specific to a certain disorder are of significant importance. For example, biomarkers for depression may enable characterization or differentiation of depressive subtypes or specific symptoms and may predict treatment response, in turn facilitating a more effective, targeted, and fast-acting approach to treatment [28]. However, we intentionally focused on stress markers to contribute to the elucidation of the mechanism of the process leading from stress to mental disorders, and to enable an objective understanding of the pre-symptomatic state of the mind.

All stressed animals exhibited reduced body weight, which was more pronounced in the chronically stressed rats (Figure 1A), suggesting increased metabolic turnover by stress. Serum corticosterone levels were augmented depending on the duration of stress (Figure 1B). These results support previous reports demonstrating that stress increases serum levels of glucocorticoids, which are rapidly produced via the hypothalamic–pituitary–adrenocortical axis, and induces metabolic effects, including release of energy substrates in the blood [26,29]. It has been suggested that elevated levels of corticosterone may result in susceptibility to oxidative/nitrosative cerebral damage [30].

Stress paradigms induce behavioral changes that might be regarded as anxiety and/or fear-like symptoms. We observed that stressed groups exhibited various anxiety-like symptoms in an open field, including decreased rearing (Figure 2A), travel distance (Figure 2B) and entering the central square (Figure 2C,D), and increased grooming (Figure 2F) and stopping time (Figure 2G), similarly to previous studies [31,32]. However, the percentage of entering the central square was significantly increased only in the 48 h stress group (Figure 2D), which may be due to the large variability of the results. Moreover, we found that water immersion stress caused a significant decrease in traveling speed (Figure 2E). These results suggest that water immersion stress reduces the migration speed, leading to a decrease in migration distance. The present analysis further revealed that the alterations in the 24 h stress group seemed maximal in entering the central square and stopping time, and changes in the 48 h stress group showed a tendency opposite to those in 12 h and 24 h stress groups. In the light/dark box test, 24 h stressed animals exhibited increased latency to enter the light room (Figure 3A), and 48 h stress reduced the time spent in the light room (Figure 3B). Rats might show anxiogenic behavior after exposure to 48 h stress, while 24 h stress may provoke an anxiolytic response. These anxiogenic symptoms may have two components: a protective response and a response to emotional disturbance. Therefore, a 24 h stress may provide a model for a pre-symptomatic state of mental disorders, and the factors identified at this time point may be stress markers critical to the development of symptoms.

In the present study, we measured the expression of 6 proteins in the hippocampus and the expression of eight genes in the hippocampus and peripheral blood. BDNF protein expression in the hippocampus was significantly decreased in the 48 h stress group (Figure 4B). Dysregulation of BDNF is associated with depression [6], and antidepressant treatments normalize BDNF expression [16]. Indeed, low levels of BDNF were reported in the hippocampus of patients with depression [33] and stressed animals [34]. The present results support the hypothesis that BDNF may be a suitable marker for chronic stress, which causes depression. However, 12 h and 24 h stressed rats exhibited no significant change in BDNF (Figure 4B), while anxiogenic behavior was observed in the open field test at these time points (Figure 2A–D,F). Taken together, BDNF may be a suitable marker for highly stressed conditions, i.e., a marker of depression, but may not be suitable for the evaluation of stress levels at an early stage.

PRX3 protein expression was significantly decreased in the hippocampus after 48 h water immersion stress (Figure 4C). PRX6 protein expression tended to be low in the 48-hour stress group (Figure 4D). PRX3 and PRX6 are associated with hippocampal atrophy and with striatal neurodegeneration in Parkinson’s disease [35]. It was demonstrated that CMS resulted in oxidative damage in adult brains, including the hippocampus, striatum, and cortex [36], which agrees with observations in patients with depression [37]. Thus, our results suggest that CMS may decrease the expression of PRX3, one of the antioxidant proteins in rat brain. The fact that the decrease in PRX6 by 48 h stress was not statistically significant may be attributed to the large variability of gene expression levels, as shown in Figure 4D. Differences should be clarified in stress responses between PRX3 and PRX6 in the future.

In the present study, we measured gene expression levels by combined RT-PCR and gel electrophoresis. The conventional RT-PCR method followed by gel electrophoresis has recently been described as semi-quantitative, whereas real-time RT-PCR is characterized by RT quantitative PCR. However, conventional RT-PCR can ensure quantitative performance depending on the method. A disadvantage of RT-PCR is that gene expression levels are saturated by the high PCR cycle number. Therefore, we clarified the PCR cycle number at which the expression level was saturated for each gene and employed the PCR cycle number slightly below the saturation in the present study. The linearity between the amount of cDNA and its OD was confirmed by a dilution series of cDNA in each sample. We verified that the ODs obtained from each sample were within the linearity range confirmed in preliminary experiments.

We observed an increased expression of *MKP-1*, *CEBPD*, *SENP5*, *MMP-8*, and *KSR1* in the hippocampus after 12 h and/or 24 h stress (Figure 5B–F). The 48 h stressed animals showed increments in *MKP-1*, *CEBPD,* and *MMP-8,* with reductions in *KLF4*, *LEF1,* and *NGFR* (Figure 5G–I)). MKP-1 is a negative regulator of the MAPK cascade, which regulates cell growth and proliferation, differentiation, neural plasticity, inflammatory responses, and apoptosis. It has been suggested that MKP-1 is a key factor in the pathophysiology of major depression [4]. Oxidative stress may regulate the expression of MKP-1 in the rat dorsal medulla [38]. CEBPD, a member of the C/EBP transcription factor family [39], is regulated by the cAMP response element-binding protein (CREB), which is involved in the formation of long-term memory and the inflammatory response [40]. CEBPD has been particularly implicated in the regulation of a wide range of genes involved in the acute phase response, including cytokines such as interleukin (IL)-1, IL-6, and tumor necrosis factor-alpha (TNF-alpha), as well as inflammatory mediators and oxidative stress factors. CEBPD may play a role in neurological disease [41] and Alzheimer’s disease [42], possibly because of CEBPD-mediated regulation of inflammatory processes. The fact that *MKP-1* and *CEBPD* expression showed a significant increase as early as 12- and 24-hour stress strongly suggests that these factors may be suitable for the evaluation of stress levels at an early stage.

SENP5 mediates both the processing and deconjugation of SUMOs, which regulate multiple biological processes, including gene expression and genome maintenance [43]. SUMO modification is involved in conditions ranging from neurodegeneration to diabetes mellitus and inflammation [43]. MMP-8 is a member of the MMP family, which is closely related to Zn^2+^-dependent endopeptidases that degrade components of the extracellular matrix. MMP-8 is associated with diverse inflammatory diseases such as neuroinflammation, multiple sclerosis, arthritis, and encephalomyelitis [44,45]). KSR1 is an essential scaffolding protein that coordinates the assembly of MAPK cascade components and controls the intensity and duration of extracellular signal-regulated kinase (ERK) activation. The absence of KSR1 leads to deficiencies in ERK-dependent synaptic plasticity and hippocampus-dependent memory formation in mice [46]. The increased expression of these three genes in the present study, from the early stage of stress exposure, suggests that these genes can be effective stress markers for evaluating pre-symptomatic mental status.

KLF4 is a transcription factor that regulates diverse biological functions, including apoptosis, proliferation, and differentiation [47]. KLF4 may function as a transcriptional suppressor of axonal growth in regenerating retinal ganglion cells and in other neurons of the central nervous system [48]. LEF1/T cell factor (TCF) is a member of the TCF family of transcription factors and binds to Wnt signaling-responsive elements. The Wnt signaling pathway may activate genes involved in multiple cellular and developmental processes [49] and regulate adult hippocampal neurogenesis [50]. NGFR binds all members of the neurotrophin family. In the adult brain, NGFR is primarily expressed in cholinergic neurons of the basal forebrain, a subset of which project to the hippocampus from the medial septum. NGFR signaling may interact with the cholinergic system to regulate anxiety-like behaviors and the acute stress response [51]. The expression of the above three kinds of genes was significantly lowered by 48 h stress in the present study. Therefore, these genes may be usable as a stress marker after the influence of stress becomes severe, similar to BDNF.

Among these factors, 12 h and/or 24 h stress caused an increase in protein expression of MKP-1 and MMP-8 in the hippocampus (Figure 4E,F). MKP-1 was also induced after 48 h stress (Figure 4E). These changes were similar to those observed with *MKP-1* and *MMP-8* gene expression, suggesting that stress induced changes at both a transcriptional and post-translational level. However, the protein expression of MMP-8 in the 48-hour stress group did not significantly differ from that in the control group, and the MMP-8 gene expression level did not change in the 12-hour stress group. Such temporal lag in genes and proteins has to be investigated further. We also found alterations in *NGFR* expression levels in the rat hippocampus. Loss of NGFR, which is a low-affinity receptor for BDNF and primarily expressed in basal forebrain cholinergic neurons, increases anxiety-like behavior in mice [52]. Therefore, we evaluated NGFR protein expression to investigate the regulation of neurotrophic signaling. Animals after 24 h and 48 h stress exhibited decreased levels of NGFR (Figure 4G). Although no changes were observed in BDNF levels after 24 h stress (Figure 4B), these changes in NGFR levels could increase BDNF-TrkB signaling by suppressing BDNF-NGFR signaling, resulting in an increase in apparent anxiogenic behavior, which could be regarded as resistant behavior in the open field test in the 24 h stressed rats. However, loss of BDNF in 48 h stressed rats could lead to reduced BDNF-TrkB signaling and elicit depressive symptoms in the open field test and light/dark box test. We, therefore, suspect that the anxiety-like behaviors in the 12 h and 24 h stressed animals might be protective reactions in response to aversive stimuli.

In the peripheral blood, increased expressions were found in *MKP-1* and *CEBPD* in 12 h, 24 h, and 48 h stress groups (Figure 6B,C). *MMP-8* expression levels were also augmented after 24 h and 48 h stress but not after 12 h stress (Figure 6E). On the other hand, 24 h and 48 h stressed rats exhibited a decrease in *LEF1* expression (Figure 6H). Expression of *NGFR* was significantly low after 48 h stress (Figure 6). These alterations were quite similar to those in the hippocampus. Therefore, it could be valuable to measure expressions of these factors in the blood to estimate changes in the hippocampus caused by stress. Rowland et al. [53] have pointed out the importance of blood biomarkers, such as high-sensitivity C- reactive protein/interleukin-6, BDNF/tumor necrosis factor (TNF)-alpha, and soluble TNF-alpha receptor 1, for understanding the mental state of bipolar disorder. Our results further suggest new stress markers that may be useful for assessing stress levels that lead to mental disorders.

The present results suggest that several factors that had not been indicated as stress markers might possibly be novel stress markers. Alterations were observed in the expression of five genes and three proteins in the hippocampus of animals exposed to stress for no longer than 24 h, which may be a model of pre-onset mental disorders. These genes and proteins may be useful factors for identifying stress levels to prevent the further development of symptoms. Among these, the expression of *MKP-1*, *CEBPD*, and *MMP-8* showed similar changes both in the hippocampus and peripheral blood. Although the relationship between cerebral events and alterations in the peripheral blood remains to be clarified, these three factors could be strong candidates as stress-responsive biomarkers. Moreover, due to the properties of RT-PCR, we might underestimate, but not overestimate, changes in gene expression levels. It cannot be denied that follow-up experiments using real-time RT-PCR (RT quantitative PCR) will be important in the future.

## 4. Materials and Methods

### 4.1. Animals and Stress Protocol

All experimental procedures were performed in accordance with animal experiment regulations established by Toho University (Tokyo, Japan) Animal Experiment Committee. A total of 117 young adult male Wistar rats (8 weeks old, weighing 220–250 g) were obtained from Clea Japan (Tokyo, Japan). Rats were housed in acrylic cages and maintained in a temperature- (23 ± 2 °C) and humidity-controlled environment with a 12 h light-dark cycle (from 07:00 to 19:00). Three to four rats were housed in each cage with access to food and water ad libitum. After acclimatization, 9-week-old rats were randomly divided into four groups: one group was subjected to a conventional environment without stress (control group, 38 animals), and the others to a stressful environment for 12 h (24 animals), 24 h (33 animals), and 48 h (22 animals). In stress groups, cages were filled with water to a depth of 2.0 cm. In this environment, the limbs of each rat were constantly underwater, which may lead to sleep deprivation (water immersion stress). Tap water and laboratory chow were available ad libitum in all experimental conditions (stressed and control).

### 4.2. Sample Collection

Brain and peripheral blood samples were collected from rats not subjected to behavioral analysis after the stress challenge in order to avoid possible stress due to behavioral analysis. After each specified time under the conditions described above, rats were decapitated, and their brains were carefully removed and placed on ice. Hippocampus was immediately dissected and quickly immersed in liquid nitrogen. Tissue samples were ground in liquid nitrogen, and the fine powders were stored at −80 °C until used for protein and gene expression analyses. Rat blood was centrifuged, and pellets (blood cells) and supernatants (serum) were preserved at −80 °C until used for corticosterone and gene expression analyses.

### 4.3. Open Field Test

Open field tests were performed in both control and all stressed groups (12 h, 24 h, and 48 h) after rat body weight was measured with a gauge. After completion of the stress paradigm, stressed animals were dried in a non-stressed environment (40–50 min). Rats were placed in the central square of a plastic-circular container (60 cm × 60 cm × 35 cm high) in a brightly lit room. The apparatus was equally divided into 13 cm × 13 cm squares (9 parts) on the floor of the arena. The remaining regions were divided into 12 areas by drawing extension lines for each square. In addition, a container was separated into two zones: the central (inside) zone (21 cm in diameter) and the outside zone (the rest of the area). In each 5 min session, the following measurements were recorded: defecation and urination frequency, traversal distance (movement of the lower hind leg into an adjacent lattice), number of times an animal entered the central square, rearing frequency (a sustained posture of hind paws placed on the floor with front paws raised or touching the walls), grooming frequency (including washing or mouthing of face, forelimbs, hind paws, body, genitals, or tails), times entered the central square as a percentage of locomotor activity ((the number of times an animal entered the central square) / (the number of locomotor activities) × 100), stopping time ((time spent immobile) + (time spent grooming) + (time spent rearing)), locomotor activity (300 s — (stopping time)), time spent in the outside zone, and traversal speed ((traversal distance) / (locomotor activity time) × 60 s). All tests were performed between 09:00 and 12:00, and all behaviors were recorded using a video camera. The arena was cleaned after every test session. Each analysis was repeated 3 times on different days, and the mean value was used as the value for one individual. A series of behavioral analyses on 4 groups were performed twice using different individuals.

### 4.4. Light/Dark Box Test

After measuring the body weight of rats, light/dark box tests were conducted for both control and stressed animals. A plastic box (66 cm × 44 cm × 32 cm) was equally divided into an exposed (light) room and an enclosed (dark) room. The entrance between the two compartments measured 8 cm × 10 cm. Stressed rats were dried, as above, before the test was performed. Rats were initially placed in the brightly lit room and allowed to freely explore the apparatus. During each 5 min session, rats were scored manually for the following parameters: frequency of crossing the entrance, latency to enter the light room (initial placement of the lower forelimbs into the entrance), total time spent in the light room (placement of lower forelimbs into the entrance), and time spent in the light room ((total time spent in the light room) — (latency)). All tests were performed between 09:00 and 12:00, and all behaviors were recorded using a video camera. The arena was cleaned after every test session. Each analysis was repeated 3 times on different days, and the mean value was used as the value for one individual. A series of behavioral analyses on 4 groups were performed twice using different individuals.

### 4.5. Total RNA Extraction

Finely powdered brain samples were completely dissolved in QIAzol reagent (QIAGEN, Hilden, North Rhine-Westphalia, Germany). After chloroform addition, supernatants were mixed with an equivalent volume of 70% ethanol. Total RNA samples were DNase-treated with RNase-Free DNase (QIAGEN) and purified with an RNeasy Mini Kit (QIAGEN). Total RNA purity and density were measured using a spectrophotometer (NanoDrop 2000, Thermo Fisher Scientific, Waltham, MA, USA). Furthermore, formaldehyde-agarose gel (1.2%) electrophoresis (50 V, 45 min) was performed, and RNA quality was confirmed by visualization (ChemiDoc XRS Plus, Bio-Rad, Hercules, CA, USA). Total RNA was stored at −80 °C.

Blood cell samples were completely dissolved in Solution D (containing 4 M guanidium thiocyanate, 25 mM sodium citrate, 0.5% lauryl sarcosine, and 0.1 M 2-mercaptoethanol). Samples, including a 1/10 volume of 3 M sodium acetate (pH 5.5), were mixed with Tris-EDTA-phenol and chloroform. Samples were then centrifuged, and pellets collected from these solutions (together with 1 volume of propanol) were compounded in sterile distilled water and Buffer RLT (QIAGEN; containing 2-mercaptoethanol). Total RNA was also extracted using the process described above.

### 4.6. Reverse-Transcription Polymerase Chain Reaction (RT-PCR)

After validating RNA purity and quality, cDNA was subsequently synthesized, and RT-PCR was performed using a commonly used housekeeping gene, *glyceraldehyde 3-phosphate dehydrogenase* (*GAPDH*), as a positive control. Synthesis of cDNA was performed using an Affinity Script QPCR cDNA Synthesis Kit (Agilent Technologies, Santa Clara, CA, USA) with a thermal cycler (GeneAmp PCR System 9700, Applied Biosystems, Waltham, MA, USA) under conditions of 25 °C for 5 min, 42 °C for 5 min, 55 °C for 40 min, and 95 °C for 5 min. In addition, cDNA was amplified in solutions containing EmeraldAmp PCR Master Mix (Takara, Tokyo, Japan) and the relevant primers. The 3′-UTR gene-specific primers were targeted against the genes listed in Table 1. Sequences for *MKP-1*, *CEBPD*, *KLF4*, *LEF1*, *SUMO1/SENP5*, *MMP-8*, *KSR1*, and *NGFR* were obtained from the National Center for Biotechnology Information (NCBI) database. Thermal cycler parameters for PCR were as follows. After initial denaturation at 97 °C for 5 min, 25–35 cycles of 95 °C for 45 s, 55–65 °C for 45 s, and 72 °C for 1 min were performed. A final elongation step was performed at 72 °C for 10 min. PCR reaction solutions were then loaded onto a 1.6% agarose gel. Electrophoresis (100 V, 31 min or 85 V, 39 min) was performed in 1× TAE buffer, using a Mupid-ex electrophoresis system (Advance, Tokyo, Japan). Gels were stained with ethidium bromide for 10 min. Bands were then visualized using a ChemiDoc XRS Plus imaging system and quantified using Image Lab Software, version 3.0 (Bio-Rad). The intensity of all samples obtained was expressed relative to *GAPDH* expression in each sample. All assays were performed in duplicate, and the mean value was used as the value for one individual. An experimental series was performed twice using samples from different rats. When the second series was assayed, several samples measured in the first series were included to correct the absolute counts. Such measurements were repeated 3 times to ensure reproducibility. In preliminary experiments, we first clarified the PCR cycle number at which the expression level of each gene saturates. Based on these results, we adopted the PCR cycle number lower than the gene expression level saturating in the present study. We also performed RT-PCR and electrophoresis in a dilution series of the amount of cDNA in a sample containing a high amount of each gene and confirmed that the relationship between the amount of cDNA and its OD was linear. The intensities obtained from each sample were within the linear range in the preliminary experiments described above.

### 4.7. Enzyme-Linked Immunosorbent Assay (ELISA)

Rat corticosterone was detected using a competition ELISA according to the manufacturer’s instructions (DetectX Corticosterone Enzyme Immunoassay Kit, Arbor Assays, Ann Arbor, MI, USA). All serum samples were diluted 100 times with the supplied Dissociation Reagent and Assay Buffer. The color was developed using the supplied tetramethylbenzidine (TMB) substrate for 10 min, and optical density was measured at 450 nm. All assays were performed in duplicate, and the mean value was used as the value for one individual. These measurements were repeated 3 times to ensure reproducibility.

### 4.8. Extraction of Total Soluble Protein

Total protein was extracted from rat hippocampal powdered samples using a previously described lysis buffer containing thiourea and Tris (LB-TT) (Hirano et al., 2006; Seo et al., 2008). LB-TT was slightly modified as follows: 7 M (*w*/*v*) urea, 42 g; 2 M (*w*/*v*) thiourea, 15.2 g; 4% (*w*/*v*) CHAPS, 4.0 g; 18 mM (*w*/*v*) Tris-HCl (pH 8.0), 1.8 mL; 14 mM (*w*/*v*) Trizma base, 169.5 mg; 0.2% (*v*/*v*) Triton X-100, 0.2 mL; 50 mM (*w*/*v*) dithiothreitol, 771.5 mg; 1% (*v*/*v*) pH 3–10 pharmalyte, 1 mL; and two EDTA-free proteinase inhibitor tablets in a total volume of 100 mL. For protein extraction, 1 mL of LB-TT was quickly added to 2 mL microfuge tubes containing powdered samples (immediately after removal from the −80 °C freezer) and immediately vortexed (at maximum speed using a Lab mixer, Scientific Industries, NY, USA) for 1 min at room temperature (RT). The LB-TT protein solution was incubated at RT for 30 min with occasional vortexing (for 30 s), and sonication (for 30 s in a water-bath type sonicator) was performed five times. Insoluble proteins and/or debris were pelleted by centrifugation at 15,000 rpm for 15 min at 20 °C in a high-speed refrigerated micro centrifuge (MX-150, TOMY, Tokyo, Japan). Clear supernatants (around 900 mL) were transferred to new 1.5 mL microfuge tubes and stored at −80 °C as the total soluble protein. Prior to storage, a 0.2 mL aliquot of the total protein solution was transferred to a new 2 mL microfuge tube and processed for a second purification, clean up, and concentration step, using a ProteoExtract Protein Precipitation Kit (Calbiochem, Darmstadt, Germany). This additional step removes any impurities while concentrating the protein. The pelleted protein, visible as a solid white precipitate at the bottom of the tube, was re-suspended in LB-TT (approximately 0.2 mL) as above. Protein concentrations were determined with a Coomassie Plus-The Better Bradford Assay Kit (#23238, Thermo Scientific) using bovine serum albumin (BSA) as a standard and a NanoDrop 2000 spectrophotometer (Thermo Fisher Scientific).

### 4.9. Western Blot Analysis

SDS-PAGE separated proteins were transferred onto a PVDF membrane (Immobilon Transfer Membranes, IPVH304F0, Millipore, MA, USA) with Whatman chromatography paper, grade: 3MM CHR (3030-6185, GE Healthcare, Little Chalfont, UK). Proteins were transferred using an electro-transfer apparatus (Nippon Eido, Tokyo, Japan) at 81 mA for 1 h. Following the transfer of proteins onto PVDF membranes, membranes were incubated in 60 mL of 5% blocking solution (3 g skim milk powder (Difco Skim Milk powder; 232100; Becton, Dickinson and Company, Franklin Lakes, NJ, USA) in 60 mL 1× TTBS (Tween-20-Tris-buffered saline; 10× TTBS: NaCl, 80 g; 1 M Tris-HCl, pH 7.5, 200 mL; Tween-20, 5 mL)) for 1–2 h at RT. Western blotting and detection were performed using Clarity Western ECL Substrate (170-5060, Bio-Rad). The blocking solution was decanted, and membranes were washed once in 1× TTBS (5 min), followed by incubation with primary antibody solutions, including rabbit anti-beta-actin (#4967; Cell Signaling; 1:500), rabbit anti-BDNF (sc-546; Santa Cruz; 1:200), rabbit anti-PRX3 or 6 (LF-PA0030, LF-PA021; Lab Frontier; both 1:2000), rabbit anti-MKP-1 (sc-10796; Santa Cruz; 1:200), rabbit anti-MMP-8 (BS1240; Bioworld Technology; 1:1000), and rabbit anti-NGFR (sc-8317; Santa Cruz; 1:100), for 1–2 h. Membranes were then washed five times with 25 mL of 1× TTBS. After the final wash, membranes were incubated with a secondary antibody solution containing Amersham ECL anti-rabbit IgG, HRP-conjugated species-specific whole antibody (NA934; GE Healthcare; 1:10,000) for 1 h at RT. Membranes were then washed five times with 1× TTBS. For blot development, Clarity western peroxide reagent and Clarity western luminol/enhancer reagent were combined in a 1:1 ratio, applied to membranes, and incubated at RT for 5 min. The excess solution was removed, and signals were visualized using a ChemiDoc XRS Plus imaging system (Bio-Rad). Representative data from images were obtained using Image Lab Software, version 3.0 (Bio-Rad). The intensity of all samples obtained was expressed relative to beta-actin expression in each sample. All assays were performed in duplicate, and the mean value was used as the value for one individual. These measurements were repeated 3 times to ensure reproducibility.

### 4.10. Statistical Analyses

The data of rat body weight are expressed as the mean ± S.E.M. Other data are expressed as the median. Statistical significance was evaluated by Tukey–Kramer and Steel–Dwass tests. In accordance with the two-tailed probability, *p* < 0.05 was considered to be statistically significant.

## 5. Conclusions

The behavioral data suggest that 12–24 h of water immersion stress likely results in emotional instability in rats, whereas 48 h exposure may provoke the onset of mental disorders. We analyzed the expression of six proteins in the hippocampus and eight genes in the hippocampus and peripheral blood. The expression was significantly changed in animals stressed for no longer than 24 h in three proteins and five genes. Similar alterations were observed in three genes in the peripheral blood. These factors might serve as a potential stress marker. Collectively, this study enables an objective evaluation of stress and contributes to the possible establishment of protocols for the prevention of mental disorders. There have been other ways the measurement of physiological parameters to evaluate stress levels. In the present study, we obtained results to enable the assessment of stress levels in the brain by measuring biomarkers in the peripheral blood. In the future, grasping the mental state by multiple methods will lead to measures to alleviate stress.

## Figures and Tables

**Table 1:** Primer combinations used for RT-PCR.

	Forward Primer		Reverse Primer			
Accession	Primer	Nucleotide Sequence (5′–3′)	Primer	Nucleotide Sequence (5′-3′)	Product	Description
(Gene)	Name		Name		Size (bp)	
X02231 X00972	RB001	TCCCTCAAGATTGTCAGCAA	RB002	AGATCCACAACGGATACATT	308	*GAPDH*
NM_053769	KS001	GACAACCACAAGGCAGACATTA	KS002	GGGAAGTTGAAGACCGTTGTAG	347	*MKP-1*
NM_013154	KS003	ACCAGGAGATGCAGCAGAAG	KS004	GCCCAAGAAACTGTAGCAATTC	256	*CEBPD*
NM_053713	KS005	TCCAAGGGACAAAAGAAAAGAA	KS006	TGGCTTTTTAGAAGGCAAAGAG	312	*KLF4*
NM_130429	KS007	CAGAGAAAGGAACAGGAGCCTA	KS008	TTCTGGGACCTGTACCTGAAGT	319	*LEF1*
XM_002724729	KS009	GGCAGACTGCTGTTACAAAGTG	KS010	ACAATAAGTGGCCCAATACCAC	305	*SENP5*
NM_022221	KS011	CATATCTCTGTTCTGGCCCTTC	KS012	GCTATGCTAGTGGGGTAACCTG	291	*MMP-8*
NM_001108284	KS013	CAGAAGGAAGAGGAAAAGCAAA	KS014	CGGTCTAGAAGCCACAGAGATT	251	*KSR1*
XM_001080891						
XM_340852						
X05137	KS015	CTGGGTTACCAGCCTGAACATA	KS016	GCTGGCTAGAACATCAGTCGTC	249	*NGFR*

Accession numbers are derived from the National Center for Biotechnology Information (NCBI). KSR1 has three accession numbers. RB, rat brain.

## Data Availability

The data presented in this study are available on request from the corresponding author.

## Abbreviations

APP	Acute Phase Protein
BDNF	Brain-Derived Neurotrophic Factor
CEBPD	CCAAT/Enhancer-Binding Protein (C/EBP) Delta
CMS	Chronic Mild Stress
GAPDH	Glyceraldehyde 3-Phosphate Dehydrogenase
KLF4	Krüpppel-like factor 4
KSR1	Kinase Suppressor of Ras 1
LEF1	Lymphoid Enhancer-Binding Factor 1
MKP-1	Mitogen-Activated Protein Kinase (MAPK) Phosphatase 1
MMP-8	Matrix Metalloproteinase-8
NGFR	Nerve Growth Factor Receptor
PRX	Peroxiredoxin
SENP5	Small Ubiquitin-Like Modifier Proteins (SUMO)1/Sentrin-Specific Peptidase 5
Stress Marker	Stress-Responsive Biomarker
TCF	T Cell Factor
